# Not All PID is an STD: A Patient's Perspective on Dismissal, Delay, and Diagnostic Bias

**DOI:** 10.1177/23743735251383591

**Published:** 2025-09-24

**Authors:** Vaishnavi J Patel, Devki Patel, Kedar J Patel, Aakaash Duggal

**Affiliations:** 1 Department of Population Health, Division of Family Medicine, 377659 The University of Texas at Austin Dell Medical School, Austin, Texas, USA; 2 12342 Texas Tech University Health Sciences Center School of Medicine, Lubbock, Texas, USA; 3 171769 The University of Texas at Austin, Austin, Texas, USA; 4 12332 Texas A&M University Naresh K. Vashisht College of Medicine, College Station, Texas, USA

**Keywords:** pelvic inflammatory disease, patient perspective, sexually transmitted infections, patient family, support system, infection, patient-centered communication

## Abstract

Pelvic inflammatory disease (PID) is often clinically associated with sexually transmitted infections, which can overshadow a broader differential diagnosis. This patient perspective explores the emotional and clinical consequences of an emergency department visit where assumptions about sexual activity delayed appropriate treatment and resulted in family conflict. As a college student from a conservative cultural background, I was disbelieved when disclosing that I had never been sexually active, and my worsening condition was attributed to denial rather than medical nuance. This piece underscores the importance of approaching patient narratives with cultural humility and avoiding premature diagnostic closure. Recommendations are made for improving history-taking, provider training, and restoring patient trust.

## Introduction

In medical education, we are taught that “when you hear hoofbeats, think horses, not zebras”—an axiom meant to encourage diagnostic probability over possibility. We are also cautioned against cognitive pitfalls like anchoring, where an initial impression overrides new evidence, and premature closure, where diagnostic reasoning ends too early. But what happens when the assumed “horse” is not just a matter of probability, but one rooted in stereotypes and cultural bias? As a young South Asian woman raised in a conservative household, my first encounter with the healthcare system as a patient was not only physically excruciating but emotionally jarring. It exposed the subtle, yet dangerous, ways in which patient narratives can be dismissed when they challenge preconceived norms.

Pelvic inflammatory disease (PID) is an infection of the female genital tract.^1^ It affects roughly 1 million women annually in the United States, with the most common causes being chlamydia and gonorrhea.^1^ However, PID is not exclusively a consequence of sexually transmitted disease (STD). In fact, both early work and more recent studies have demonstrated that more than a quarter of PID cases are unrelated to *Chlamydia trachomatis* or *Neisseria gonorrhoeae*, though this knowledge is often underemphasized in clinical practice.^2,3^ Regardless, sources of information for patients and providers advise that PID is predominantly due to gonococcal or chlamydial infection without entertaining the other possibilities.^4^ Other causes, including procedures like intrauterine device insertions, non-STD microorganisms, or even postpartum complications, can also be culprits.^1^ Yet, these possibilities often receive less attention in research, literature, and clinically.^1^ My story highlights how this clinical tunnel vision delayed my diagnosis and eroded my faith in the system I was training to be a part of.

## Perspective

At the time, I was a junior in college, pre-med, and just 21. I had recently returned from a trip to India to visit my aging grandparents. Like other pre-meds, I was focused on grades, extracurriculars, and supporting my family financially through multiple jobs, including cashiering at my family's 7-Eleven. Dating or intimacy was not on my radar. I have always believed in waiting until marriage for physical relationships. This was not a casual value, but one deeply rooted in my upbringing and identity.

While at work one evening, I began to feel nauseous and fatigued. My thoughts felt foggy, like I was “not all there.” Soon after, I developed intense abdominal pain that made standing difficult. When over-the-counter pain meds failed, I went to the emergency department. That decision would set in motion one of the most dehumanizing experiences of my life.

In the ER (day 0), I explained my symptoms and shared my travel history. It was difficult to explain, as I was experiencing severe pain. The attending physician quickly diagnosed PID based on my physical exam findings, and let me know it was likely from an STD. I was confused. I told him I had not been sexually active—not ever. I was told to “be honest” and that they would not inform my parents or siblings, who were in the waiting room, of my sexual history. His expression shifted to suspicion, and when I insisted on my history, it was met with dismissal. After this, I became confused and unable to answer questions, so my family was tasked with filling in the blanks. Later, I came to learn that my family was also questioned about my sexual history and met with the same skepticism—all while I was still writhing in pain within the confines of my ER bed. Upon the insistence of my sister, who was determined to validate my history in the eyes of the ER doctor, they ran an STD panel, all of which came back negative. Despite this, they dismissed the results as false negatives. My family and I were told that due to my unstable vital signs requiring pressors, my new altered mental status, developing respiratory distress, and my “concerning” lab work, I would be admitted directly to the ICU.

In the ICU (day 1), my condition continued to worsen. My blood pressure remained very low despite the many pressors that I was put on, and my mental status fluctuated heavily. The next day (day 2), my respiratory status decompensated to the point of requiring a ventilator and feeding tube. My family was told to give the pressors and antibiotics that I was on “more time” to start working. When asked about the antibiotics I was on, my family was told that I was being given antibiotics for microorganisms common in PID, which are typically sexually transmitted infections, even though my history and tests suggested otherwise. Attempts at changing the antibiotics or getting further imaging were delayed to give the antibiotics more time to work. On day 6 of the hospitalization, we were told that none of the antibiotics I was being given appeared to be working and they would start a different one. During that time, my relatives were informed of my diagnosis. When they googled PID, many found that sexually transmitted infections were the most common cause. In addition to that, they were told that the antibiotics I was on were for STIs. This led to judgment and whispers among distant family members who assumed I must have been promiscuous and that this was some sort of punishment. My aunt even told my father to watch me more closely, expressing concern that I was “sleeping around” and blaming my parents’ “bad parenting” for it.

No one explained what was happening to my immigrant parents clearly. Many communications were filled with medical jargon or were not adjusted for my parent's poor understanding of the English language. At one point, my father tried asking a nurse when they would be able to take me home, to which he was coldly told, “What makes you think she's ever going home?” We had to comfort my parents all night while they wept. Nurses in the ICU came and went, rarely making eye contact due to fear of my family asking more questions of them.

As days passed in the ICU, my condition worsened. My family insisted on speaking with a different provider. It was actually the fifth physician to be added to my case, an OB/GYN who was a woman of color, who believed my initial history and explained the next steps to my family (day 7). She decided to run additional cultures and pathology beyond the standard STD panel. These additional tests also took a few days to come back. Since the management of PID is well-understood to require a certain series of antibiotics, I was kept on the same antibiotics that we were already told appeared to not be working. The chances of me making a complete recovery began to look grim. Those cultures came back confirming an unusual strain of Escherichia coli. We were told that it typically inhabits the water in India—a strain resistant to the standard antibiotics geared toward the STDs everyone had assumed I had. This discovery finally redirected my treatment, and my condition began to improve.

I was never told explicitly what the suspected cause was earlier, nor was there any acknowledgment or apology for the delay or disbelief. No one explained to my family, friends, or relatives the potential causes of PID outside of STDs. No one asked them for their understanding of a pelvic infection. No one asked me again about travel or environmental exposures. I went home with pills and paperwork after 2 more weeks in the stepdown unit, but the emotional bruising lingered far longer.

Beyond the immediate crisis, the damage from this infection left lasting consequences. The destruction and scarring to my reproductive organs, especially my fallopian tubes, has resulted in infertility. Even with potential medical intervention, I have been told that I now carry a much higher risk of miscarriage and ectopic pregnancy—a reality that continues to shape my life years after that hospitalization.

Provided below are some excerpts from the medical records:

- The ER Provider (Day 0) “Patient insisting that she has never been sexually active,” “Her Chalmydia test is negative.” “Recommended Check NAAT urine for Chlamydia/Neisseria,” “She does note some brown vaginal discharge out of the ordinary which is confirmed on pelvic exam although patient adamantly claims that she is not sexually active,” “Given that patient says she is not sexually active and therefore low risk for PID, I ordered a Computed Tomography scan of her abdomen and pelvis to evaluate for possibility of appendicitis which revealed a normal appendix but signs concerning for PID as well as bilateral ovarian cysts vs abscess and recommended pelvic US.” “I initially had ordered vancomycin/zosyn/clindamycin for broad spectrum coverage including against staph toxic shock syndrome but after CT results I have added gentamycin to cover for TOA while awaiting ultrasound results as patient appears to be getting worse and MAP around 65.” It was not mentioned explicitly in the notes but per the orders, the vancomycin and zosyn order was discontinued when the gentamicin was started.
- The CCU Provider (Day 1): “Check NAAT urine for Chlamydia/Neisseria” I was tested for chlamydia and gonorrhea 2 more times following this, despite all previous tests coming negative.
- The CCU Provider (Day 4) “patient is still having abdominal pain 7/10 this morning. Still having, tachycardia in 120 to 130, her WBC went up from 14 to 20 to 22 this morning at 3Am. Febrile overnight. No clinical improvement on ABX.”
- The CCU Provider (Day 6) “OBGYN has arrived. They recommend in addition to clindamycin and gentamicin, Flagyl which I have ordered.” “Continue gentamicin, clindamycin, Flagyl per OBGYN recommendation.”
- The CCU Provider (Day 7): “Continued fevers as high as 101” “Obstetrics joint and evaluated the patient today adjust antibiotics” “On broad-spectrum antibiotics All cultures pending.”

## Practical Recommendations

The experience I had, unfortunately, was not isolated. They were also not just clinical oversights, but symptoms of a healthcare system that too often confuses pattern recognition with patient understanding and is full of jaded providers and implicit bias.^5,6^ I discuss my perspective as a patient in hopes that it will bring awareness to these issues and encourage discussion about solutions. To mitigate healthcare situations like my own, there are concerns in the healthcare system that must be addressed, considered, and changed.

Adopting a wide lens when forming a differential diagnosis, even if that requires brushing up on some clinical knowledge, is essential. While it is widely understood that most cases of PID can be linked to STDs, up to 15% of cases are due to microorganisms that are not sexually transmitted.^7^ These less common cases can be identified through taking a careful history, including travel history, medical history, procedure history, and environmental exposures, and integrating these components into clinical decision-making.^5^ In this case, my recent travel to India was not considered relevant until after multiple delays, despite well-documented risks of antibiotic-resistant organisms in certain regions. Though this interpretation is specific to non-STD causes of PID, the same principles apply to any disease with common and less-common causes. An accurate diagnosis can be influential in guiding diagnostics and management.

In the emergency department, I was started on gentamicin and clindamycin, a regimen highly effective against Chlamydia trachomatis and Neisseria gonorrhoeae, the organisms most often linked to PID.^8^ Despite this coverage, my condition deteriorated over the next 5 days to the point that I required a ventilator and feeding tube. At that stage, only metronidazole was added and given more time to work, despite the worsening of my clinical status. When a new OB/GYN joined my team, she broadened the diagnostic evaluation with additional cultures and swabs, though the antibiotics were left unchanged given their role as first-line therapy. It was only multiple days later, when those cultures resulted, that the true cause was identified: a resistant strain of Escherichia coli not susceptible to the standard regimen. My treatment was then shifted to piperacillin-tazobactam, the only antibiotic to which the organism was sensitive, and I finally began to improve. Though it is often thought that gentamicin and clindamycin is broad-spectrum coverage for PID, the regimen is not ideal for E. coli, especially international strains.^9–11^ While sexually transmitted infections are the leading cause of PID in the United States, studies show that in India, non-sexually transmitted organisms such as Staphylococcus aureus, Klebsiella species, Escherichia coli, and Pseudomonas species account for the vast majority of cases.^12,13^ This is also demonstrated by a change in the first-line management between the 2 countries. Though gentamicin and clindamycin are always first-line in the United States due to the prevalence of STDs, antibiotic choice for PID in India is frequently done on a case-by-case basis with recommendations that do not include aminoglycosides nor macrolides.^14^ For a patient without a sexual history, in India, E. coli PID would have been at the top of the differential diagnosis list.^12,13^ Importantly, it is not reasonable to expect providers to know the microbiologic landscape of every country or region, but it is reasonable to expect that new information, such as a recent travel history, should meaningfully guide management. Recognizing when the working differential needs to be widened, rather than prematurely closed, can be essential to the care of a patient.

Cognitive biases such as anchoring—fixating on an initial diagnosis despite conflicting evidence—and premature closure—ending diagnostic reasoning too early—likely played a role in my care. These shortcuts, while often efficient, can become dangerous when they overshadow a patient's lived history or contradict objective data. Recognizing and actively mitigating these biases is essential to preventing diagnostic errors.^15^ Patients from marginalized communities are particularly vulnerable to the harms of dismissive communication and premature diagnostic closure, as these interactions can compound existing inequities in trust and access.^16^ Incorporating cultural humility into clinical encounters is therefore not optional, but essential, in mitigating these disparities and ensuring equitable care.^16^

Clinicians could greatly benefit from carefully and intentionally practicing patient-centered communication. Using verbiage that acknowledges and validates a patient's experience while still assuring clinical thoroughness such as “I believe you, but I would still like to [insert lab test, imaging modality, physical exam] just to be thorough,” “Can you tell me about your experience with [insert sensitive topic]?,” or “I believe you, let's move forward with [insert next steps]” can significantly impact patient satisfaction and trust.^17,18^ Even when a patient's honesty is in question, research shows that dishonesty often stems from understandable or justifiable reasons.^18^ Maintaining patient-centered communication remains essential to preserving trust and integrity in the doctor–patient relationship. How providers communicate can significantly affect patient experience, even when the clinical outcome does not change. A simple acknowledgment or apology, such as “Thank you for your honesty, and I’m sorry this took longer than it should have” or “I apologize, I was wrong,” can go a long way in maintaining a patient's positive narrative of their medical situation. Studies have supported that open communication improves adherence to treatment plans and reduces litigation.^17^ In this way, communication is less about information transfer and more of a clinical tool. Improving communication also requires institutional support. Continuing medical education courses, hospital-based workshops, and simulation programs that focus on bias reduction and patient-centered communication have been shown to strengthen provider skills and reinforce best practices.^19–21^ Embedding these opportunities into routine training can help sustain meaningful change at both the individual and system level.

My experience also highlighted the value of physician diversity. When I was finally seen by an OB/GYN who shared aspects of my cultural identity, my story was believed and my care shifted. There was also a benefit to this OB/GYN having had part of her training in Syria, as she was more familiar with non-STD causes of PID, leading to her having a much broader differential diagnosis for my case. A diverse physician workforce not only brings broader clinical perspectives but also fosters patient trust, improves communication across cultural backgrounds, and may help reduce the risk of misdiagnosis rooted in assumptions.^22^ Diversity has been shown to improve communication, increase trust, and reduce disparities in care—a value that my experience made deeply tangible.^23,24^

Finally, the most underemphasized recommendation across medicine is to consider the importance of provider-family dynamics. It is common for family-provider communications to be considered significant in end-of-life situations or when the patient is pediatric.^25^ However, cultural and personal influences also play a role in how important it is for a family to be part of a patient's care.^18,25^ A patient may consider their support system to be spirituality, mental wellbeing, their loved ones, or some combination of these things. Studies have shown significant benefits to family involvement in patient care, such as contribution to decision-making, assisting in care, and addressing expectations of the patient.^26^ A major downfall of family involvement, according to some studies, is the challenge it can pose to providers when faced with conflicts or dissatisfaction.^26^ However, these challenges can be overcome. It is important for providers to ensure that the support systems of patients are taken care of, as well as the patient. Being South Asian, family is an essential part of my support system, and their opinions mattered in every aspect of my care. In my situation, attention to my family would have served to reduce stress regarding my medical situation, prevent misinformation, and empower my family to assist with medical decision-making.

## Conclusion

That hospital stay left more than a scar on my medical record; it fractured my trust in a system I had once idealized. It taught me that the intersection of culture, medicine, and human experience is not always navigated with grace. It also highlighted the danger of cognitive biases, such as anchoring and premature closure, that can distort diagnostic reasoning when layered with implicit assumptions. My experience further underscored the importance of cultural humility and the value of a diverse physician workforce, both of which are essential for building trust and reducing disparities in care. As I move forward on my path to becoming a physician, I carry this memory not as a weight but as a reminder that our clinical decisions are only as good as our willingness to listen, believe, and care. Humanism in healthcare is not an elective; it is the core curriculum.
